# Prussian Blue: A Safe Pigment with Zeolitic-Like Activity

**DOI:** 10.3390/ijms22020780

**Published:** 2021-01-15

**Authors:** Joan Estelrich, Maria Antònia Busquets

**Affiliations:** 1 Department of Pharmacy, Pharmaceutical Technology and Physical Chemistry, Faculty of Pharmacy and Food Sciences, University of Barcelona, Avda., Joan XXIII, 27–31, 08028 Barcelona, Spain; mabusquetsvinas@ub.edu; 2 Institute of Nanoscience and Nanotechnology, University of Barcelona, Avda., Diagonal 645, 08028 Barcelona, Spain

**Keywords:** mesoporous adsorbents, cesium removal, thallium removal, isotherm models, kinetics

## Abstract

Prussian blue (PB) and PB analogues (PBA) are coordination network materials that present important similarities with zeolites concretely with their ability of adsorbing cations. Depending on the conditions of preparation, which is cheap and easy, PB can be classified into soluble PB and insoluble PB. The zeolitic-like properties are mainly inherent to insoluble form. This form presents some defects in its cubic lattice resulting in an open structure. The vacancies make PB capable of taking up and trapping ions or molecules into the lattice. Important adsorption characteristics of PB are a high specific area (370 m^2^ g^−1^ determined according the BET theory), uniform pore diameter, and large pore width. PB has numerous applications in many scientific and technological fields. PB are assembled into nanoparticles that, due to their biosafety and biocompatibility, can be used for biomedical applications. PB and PBA have been shown to be excellent sorbents of radioactive cesium and radioactive and nonradioactive thallium. Other cations adsorbed by PB are K^+^, Na^+^, NH_4_^+^, and some divalent cations. PB can also capture gaseous molecules, hydrocarbons, and even luminescent molecules such as 2-aminoanthracene. As the main adsorptive application of PB is the selective removal of cations from the environment, it is important to easily separate the sorbent of the purified solution. To facilitate this, PB is encapsulated into a polymer or coats a support, sometimes magnetic particles. Finally, is remarkable to point out that PB can be recycled and the adsorbed material can be recovered.

## 1. Introduction

A zeolite is a crystalline, microporous, hydrated aluminosilicate mineral having an infinite, open, three-dimensional structure. They have a framework structure that encloses interconnected cavities occupied by large metal cations (Na^+^, K^+^, Ca^2+^, Mg^2+^, and others) and water molecules. These positive ions are rather loosely held and can readily be exchanged for others in a contact solution. Zeolites are further able to lose and gain water reversibly without change of crystal structure. The large structural cavities and the entry channels leading into them contain water molecules, which form hydration spheres around exchangeable cations. On removal of water by heating at 350–400 °C, small molecules can pass through entry channels, but larger molecules are excluded—the so-called ‘‘molecular sieve’’ property of crystalline zeolites [1]. In the last 60 years, the study and characterization of zeolites has been an important factor for industrial progress, given their catalytic properties and their high adsorption and desiccant capabilities, permitting oil refining, industrial gasses treatments, industrial ovens, and industrial waste treatments. Another application is water remediation, consisting of the removal of cationic pollutants such as ammonium as adsorbents in pollution control or the handling and storage of nuclear wastes [2]. 

Prussian blue (PB) and their analogues (PBA) present important similarities with zeolites in relation its adsorption ability of cations. PB and PBA are widely recognized as the first coordination network materials in which transition metal ions are assembled through cyano-bridges, generating a 3D cubic structure. PB is a mixed-valence system exhibiting the general formula A_1−x_Fe^III^[Fe^II^(CN)_6_]_1−x_/_4_□_x/4_, in which A is a ion (K^+^, Na^+^ or NH_4_^+^) and □ denotes the hexacyanoferrate vacancies [3]. PBA are coordination polymers with the general formula A_x_M_y_[M’(CN_6_)]_z_, where A is an alkaline metal cation and M and M’ cations in oxidation state +2 or +3 [4]. Depending on the exact stoichiometry and conditions of preparation, PB can be divided into insoluble PB and soluble PB [5]. The zeolitic-like properties of PB are inherent to insoluble form. Some defects exist in insoluble PB due to the interstitial water molecules, which could be divided into two types according to their coordination: the water molecules coordinated to Fe (II) sites (coordinative water) and the ones inside cavities which do not coordinate to metal sites (zeolitic or crystallized water) [6]. Both kinds of water can be distinguished by IR: the absorption peak at 3630 cm^−1^ is characteristic of O‒H group of coordinated water, whereas the peak at 3400 cm^−1^ is derived of O‒H group of crystallized water [3]. Throughout the decades, PB has been recognized by scientists to be an inorganic coordination complex with great application potential in many fields, such as electrochromic displays, electrocatalysts, secondary batteries, ion- and biosensors, photomagnets, and hydrogen storage [7]. PB are assembled into nano-sized architectures through cyanobridged ligands, the PB nanoparticles. Currently, the synthesis of PB at the nanoscale has a great interest due to several properties arising from their molecule-based nature: solubility, stability, flexible molecular structure, porosity, low density (~1.8 g cm^−3^), and adjustable physical and chemical properties [8]. The development of PB nanoparticles has involved an upturn of their use in a myriad of biomedical applications [9]. Such nanoparticles benefit from an excellent biocompatibility and biosafety since the insoluble form of PB was approved by the Food and Drug Administration (FDA) as material with good safety for human use. Moreover, it is found in the List of Essential Medicines of the World Health Organization [10].

In this review, we will focus on the zeolitic-like properties of PB and analogues. We will highlight the applications of PB and PBA as adsorbents of cations: antidotes and binders of cations, among others. 

## 2. Synthesis of Insoluble Prussian Blue

The insoluble PB can be prepared by precipitation from aqueous solution at moderate temperatures. Two methods can be used: the direct method and the indirect one [11]. In the direct method, PB is prepared by either combining at room temperature and a rate of 1–10 mL·h^−1^ an aqueous solution of iron (III) salt, such as FeCl_3_·6H_2_O, with an aqueous solution of a ferrocyanide salt, such as Na_4_[Fe(CN)_6_]·10H_2_O or K_4_[Fe(CN)_6_]·3H_2_O, or combining an aqueous solution of iron(II) salt, such as FeCl_2_·4H_2_0, with an aqueous solution of a ferricyanide salt, such as Na_3_[Fe(CN)_6_] or K_3_[Fe(CN)_6_]. In the indirect method, Berlin white, Fe_2_[Fe(CN)_6_], is first prepared by combining an aqueous solution of an iron (II) salt such as FeCl_2_·4H_2_0, with an aqueous solution of a ferrocyanide salt, such as Na_4_[Fe(CN)_6_]·10H_2_O or K_4_[Fe(CN)_6_]·3H_2_O. Berlin white is subsequently oxidized to form the final insoluble PB. In both methods, after addition, the mixture was stirred 1 h before being centrifuged for 15 min. The supernatant was removed and the nanoparticles of PB were washed successively with water and ethanol and dried under vacuum to obtain a dark blue powder. 

The direct method is the most conventional. A modification of the direct method, so-called single-precursor method, is based on the use of [Fe(CN)_6_]^4−^ or [Fe(CN)_6_]^3−^, which can slowly release ferric or ferrous ions that are reduced or oxidized into ferrous or ferric ions, respectively, in acidic conditions, and that then react with the unreacted ions. When the coprecipitation is carried out at high temperature and pressure, the variant constitutes a hydrothermal method. 

A detailed description of other ways for preparing PB nanoparticles is found in the review of Da Carro et al. [12].

## 3. Characteristics of Prussian Blue

### 3.1. Structural Characteristics

**Figure 1:**
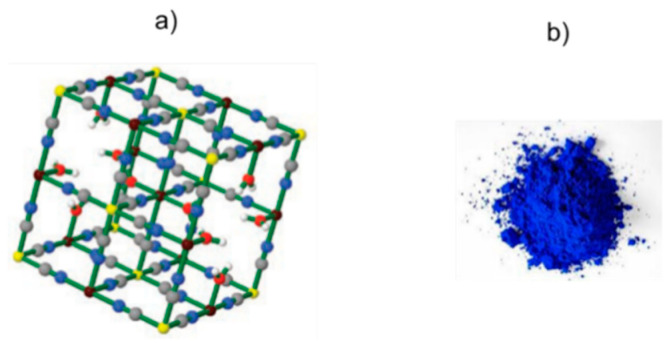
(**a**) Cubic lattice of insoluble Prussian blue. Colors: Fe (II) yellow; Fe (III) violet; C gray; N blue; O red; H white. (**b**) Visual aspect of powder of Prussian blue. Figure 1a reproduced with permission from [5].

The ideal formulation of insoluble PB is Fe^III^_4_[Fe^II^(CN)_6_]_3_·xH_2_O, where the extent of hydration, x, can vary from 10 to 16 (the exact value can be easily determined by thermogravimetric analysis (TGA)). PB is an aggregated form of ~10 nm nanoparticles [13]. The real crystal structure has been determined by X‒ray [14,15] and neutron [16] diffraction studies. PB has a cubic lattice with a face-centered cubic unit cell alternating Fe^2+^ and Fe^3+^ cations bridged by cyanide. Fe^3+^ ions are connected to nitrogen atoms of cyanides, and Fe^2+^ are linked by carbon atoms of cyanides, which alternately coordinate to form a cubic unit cell. A quarter of the [Fe(CN)_6_]^4−^ complexes must be absent and some N‒sites around Fe^3+^ are occupied by water molecules (Figure 1a). The Fe^2+^ cations are octahedrally coordinated by six carbon atoms of the cyanide anion (CN^−^) that acts as a bridge with Fe^3+^ cations that are octahedrally coordinated by 4.5 nitrogen atoms plus 1.5 oxygen atoms. In the lattice, Fe^2+^ and Fe^3+^ have different spheres of coordination, and, in consequence, they can adopt several oxidation states and magnetic configurations. 

The X‒ray diffraction pattern shows relatively strong peaks at 2θ values of 17.6°, 24.8°, 35,2°, 39.6°, and 43.5°. These peaks are ascribed to the (200), (220), (400), (420), and (422) planes of PB, which is generally claimed as the space group F3m3, although really is Pm3m [3]. The dimension of the unit cell is 1.02 nm, corresponding to the length of Fe^3+^‒NC‒Fe^2+^‒CN‒Fe^3+^, and the average bond lengths of Fe^2+^‒, C‒N, and Fe^3+^‒N=C are 0.190, 0113, and 0.203 nm, respectively [15]. 

### 3.2. Physicochemical Characteristics

PB presents an intense blue color to which its success as a blue pigment used by artists is attributable [14]. The color of PB is due to charge transfer (CT) transition of one electron from the iron (II) centers to the iron (III) centers. The wavelength of the absorption maximum of PB in water is around 700 nm. Although PB is insoluble, the mixing of a little amount of powder with water and subsequent dispersion by ultrasound bath afforded a blue suspension. Upon strong light exposure, or in basic conditions, Fe(III) ions are reduced to Fe(II), the charge transfer does not take place, and the reduced compound is colorless (this compound is known as Prussian white). The IR spectrum of PB in KBr affords absorbance at the following wave numbers: ν (O‒H) = 3630 cm^−1^ (coordination water), ν (O‒H) = 3400 cm^−1^ (crystallized water), ν (C ≡ H) = 2082 cm^−1^ (Fe(III)‒C ≡ N‒Fe(II)), δ (O‒H) = 1606 cm^−1^ (crystallized water), ν (Fe(II)‒CN) = 602 cm^−1^, δ (Fe(II)‒CN) = 501 cm^−1^ [2]. PB shows a ferromagnetic behavior below the Curie temperature (T_C_ = 5.5 ± 0.5 K) [17]. 

## 4. Adsorption Properties of PB 

**Figure 2:**
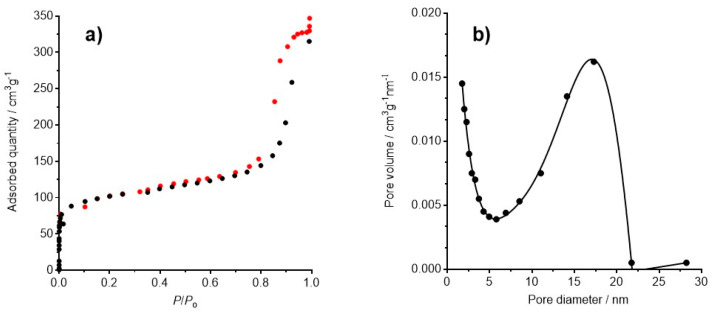
(**a**) Adsorption–desorption isotherm for BET surface area measurement. The hysteresis loop appearing at high *P*/*P*_o_ is characteristic of a type IV isotherm; (**b**) pore diameter.

The open structure of the crystal lattice of PB makes it capable of taking up and trapping other molecules or ions in the cavities of its lattice. The surface area analysis using N_2_ adsorption and desorption measurements of PB samples at 77.350 K afford significant information about the porosity, BET (Brunauer–Emmett–Teller) specific surface area, pore size, and pore volume. The size and morphology of PB nanoparticles influences the adsorptive properties. The small- (~20 nm) and medium-sized particles (around 100 nm) exhibited much higher BET surfaces areas than the large ones (in excess of 200 nm), probably due to the hindrance of removing water molecules during degassing caused by the larger size of the particles and dense crystal structure. In addition, the medium-sized particles showed the highest porosity [18]. The insoluble PB synthesized by us has a specific surface area of 370 m^2^ g^−1^, and its pore volume is 0.537 cm^3^ g^−1^. The adsorption–desorption isotherm is showed in Figure 2. This isotherm belongs to IV-type isotherm (Figure 2a) according to the IUPAC classification [19], since a distinguishable hysteresis loop appears at high relative pressure (P/P_o_ > 0.8), which demonstrated that PB is essentially of mesoporous structure. The pore diameter distribution is centered at 17.3 nm.

Due to uniform pore diameter, large pore width, and good surface area, PB and PBA are capable of hosting small molecules (water, gases, hydrocarbons) and ions (metal cations) in their crystal lattice spaces. The adsorption ability of cations by PB is influenced by the Stokes radium of the hydrated ion. Some Stokes radii are found to be smaller than the corresponding crystal radii, contrary to expectation for hydrated ions [20]. For instance, the Stokes radii of the alkali ions are Rb^+^ (0.118 nm), Cs^+^ (0.119 nm), K^+^ (0.125 nm), and Na^+^ (0.184 nm). The adsorption ability is quantified by the adsorption capacity defined as the amount adsorbed expressed in mg by the amount of PB in g. Besides the adsorption ability, the adsorbent capacity can be expressed in terms of the distribution coefficient (K_d_). The comparison among the different PB-composites is difficult since the obtained values are variable. They depend on the different morphology of the PB-composites, the temperature of mixing, the time of stirring of the solution with the sorbent (equilibrium time), the pH, the ratio ion/sorbent, etc. Finally, it is necessary to perform a reusability test.

### 4.1. Adsorption of Cesium 

First, the Chernobyl nuclear reactor disaster on 26 April 1986, and then the tsunami that caused the Fukushima nuclear accident on 11 March 2011 triggered a massive release of radioactive elements into the environment. The contamination by cesium isotopes, such as ^134^Cs and ^137^Cs, was one of the major environmental concerns. Whereas the half-live of the ^134^Cs isotope is 2 years, the radioisotope ^137^Cs has a long half-life (30.17 years). Cs is a strong emitter of gamma rays, which is a serious threat to health since due to its high solubility, high mobility, and easy incorporation into living organisms (it behaves similarly to K^+^ and Na^+^ in the biological behavior profile). Cs^+^ may cause various human diseases. To date, methods such as chemical precipitation, extraction, volatilization, adsorption, and ion exchange have been proposed for cesium removal [21]. Adsorption and ion exchange both seem to be the most simple, economical, and effective techniques. One of the most important sorbents developed for the selective removal of Cs^+^ are the nano- and microparticles formed by PB or PBA. As indicated previously, PB is an FDA-approved drug used in clinics for the treatment of radioactive exposure. The drug (trade name Radiogardase) consists of 500 mg of PB in gelatin capsules. The dose for adults is 3 g (six capsules) taken orally three times a day. The drug works by trapping Cs in the intestine, so that they can be passed out of the body in the stool rather than be readsorbed [22].

The proton/cation exchange, surface adsorption, and mechanical trapping within the crystal structure are the main mechanisms for decontamination of Cs by PB. For soluble PB, it has been reported that Cs^+^ are adsorbed on PB via ion exchange with K^+^ that are present in the cubic lattice of PB [23]. However, the Cs^+^ sorption mechanism by the insoluble PB-based sorbents is not still elucidated. It is likely that the mechanism is composed by various independent phenomena: ion exchange, physical cesium ion entrapment, and physical sorption. The physical adsorption would imply the physical sorption of hydrated Cs^+^ into the regular lattice spaces surrounded by the Fe^2+^‒CN‒Fe^3+^ bonds. The ion-exchange mechanism needs the lattice to present defect sites, where Cs^+^ is chemically adsorbed by means of proton exchange with proton elimination from the coordination water molecules [24]. The contribution of large defects in the adsorption of Cs^+^ ions has also been demonstrated in PBA [25]. Takahashi et al. have deduced that the Cs adsorption by KCu[Fe(CN_6_) was governed by three mechanisms: (a) mainly ion exchange between Cs^+^ and K^+^, (b) percolation of Cs^+^ cations through vacancy sites from the surface, and (c) proton exchange with Cs^+^ at the range of low K^+^ incorporation [26].

The pioneering employment of PBA for cesium decontamination was proposed around twenty years ago by Harjula et al. using granular potassium cobalt hexacyanoferrate (K_2_[CoFe(CN)_6_] [27]. Ishizaki et al. verified that the adsorption capacity of insoluble PB was higher than that of soluble PB [24]. As indicated in the Introduction, the coordination water molecules are common in the structure of insoluble PB, and this could be the reason of this different adsorption ability between both types of PB. Ishizaki et al. observed that the adsorbed Cs^+^ ions in PB were distributed homogenously along with the Fe ions throughout the PB nanoparticles according to the EDS mapping obtained from scanning transmission electron microscope images (STEM_HAADF images) using a high-angle annular dark field (HAADF) detector. Moreover, the almost totality of performed kinetic studies showed that the adsorption can be described by a pseudo-second-order kinetic model; this implies that the chemical adsorption process is the rate-limiting step, and that Cs^+^ are adsorbed through chemisorption rather than physical adsorption [24]. However, Fujita et al. proposed that the external surface of PB crystal was the only bonding site for Cs^+^ because of the extremely low intracrystalline diffusion coefficient (less than 3.3 × 10^−22^ m^2^ s^−1^) [28]. Due to this slow adsorption rate, the Cs^+^ could penetrate only 1–2 nm (1–2 units of the crystalline lattice) after 2 weeks at room temperature. 

Fujita et al. also demonstrated the high selectivity of PB; even when molar concentration of H_3_O^+^ was more than 200 times higher or molar concentration of K^+^ was more than 50,000 times higher than that of Cs^+^ in the aqueous solution, the equilibrium adsorption amount was reduced by only approximately one-half to two-third of that the pure system [28]. Despite the high selectivity of PB and PBA toward Cs^+^, when the sorbent is in the form of fine powder, it can produce problems at the moment of separating the sorbent from the purified solution, since fine particles can form a dense cake, which is difficult to filter. Moreover, the colloidal character of a PB suspension prevent easy separation after use. These shortcomings can be overcome by the synthesis of composites in which PB is encapsulated into a polymer matrix or located coating a support. In this way, PB or PBA were linked to a matrix formed by silica or glass [29]. The adsorbents were also incorporated into a crosslinked, bead-type copolymer resin composed of polystyrene (PS) and divinylbenzene (DVB) [30]. PB has also been immobilized by means of a linkage of polydopamine (PD) on the surface of nanofibers fabricated by electrospinning of polyacrylonitrile (PAN) [31], on poly(vinyl alcohol) (PVA) sponge [32,33], caged in a gel of sodium alginate [34], in the same colloid to develop an intestinal release delivery system [35], in spongiform adsorbents [36], bonded to sepiolite (hydrated magnesium silicate) [37], embedded into a binder matrix [38], in porous cellulose aerogel [39] or in microporous carboxymethyl cellulose nanofibrils membranes [40]. Hayashi et al. found that PB/PBA precipitated spontaneously in agarose gels, and these materials were able to adsorb Cs^+^ ions effectively [41]. Zhang et al. prepared a clay-based composite hydrogel containing PBA nanoparticles (potassium copper hexacyanoferrate). The hydrogel presented a maximum adsorption capacity in water of ~173 mg/g [42].

A good strategy to separate the contaminate product from the water is to coat PB on superparamagnetic nanoparticles. The magnetic characteristics of this core/shell nanocomposite make suitable the use of an external magnet to separate the contaminated nanoparticles [43,44,45,46,47] In this way, Jang et al. prepared magnetic nanoparticles in the presence of poly(diallyldimethylammonium chloride) (PDDA). Then, the hexacyanoferrate (II) ions react with the ferric ions released from magnetic nanoparticles (single-precursor method) [48]. However, PDDA has no superparamagnetism and Cs affinity, and thus reduces the magnetic and adsorption ability of PB-coated magnetic adsorbents. Yang et al. have reported the preparation of PB-functionalized magnetic nanoclusters. The magnetic nanoclusters were synthesized using the hydrothermal method and were then coated with PB by the single-precursor method [49]. With this method, Qian et al. prepared PB-coated magnetic nanoparticles decorated with polyethylene glycol (PEG) to remove Cs from blood [50]. Wang et al. have prepared magnetic microparticles of PB. The microparticles coated an electrode and were used as adsorption/desorption materials in the electrochemically switched ion exchange (ESIX) technique [51]. This is an environmentally friendly, time-saving, and efficient ion extraction technique that has been proposed for the extraction of target ions in trace amounts electrochemically regulating the redox state of electroactive film to control ion adsorption and desorption processes reversibly [52]. Table 1 summarizes the most relevant adsorption properties of magnetic PB structures.

An original, although rather complex, way to adsorb Cs^+^ has been reported by Kohiyama et al. [53]. They prepared extruded liposomes hydrating the lipid with a hexacianoferrate (III) salt. Then, the liposomes were treated with the antifungal polyene antibiotic amphotericin B (AmB) and with Mohr’s salt ((NH_4_)_2_Fe(SO_4_)_2_·6H_2_O). The salt entered the aqueous inner of the liposome through the channels formed by AmB in the bilayer and PB was formed inside the liposome. As main conclusion, the authors pointed out that the liposomes showed higher Cs^+^ adsorption capacity than the PB nanoparticles in aqueous media.

In order to enhance the adsorption properties of PB composites, usually formed by nanoparticles with cubic morphology, a three-dimensional hierarchical PB constructed by interconnected ultrathin nanosheets was prepared by Bu et al. [54]. Nitrogen sorption analysis showed a drastic increase in the nitrogen adsorption–desorption isotherm of 3D hierarchical structures in comparison to cubic PB microcrystals. These results may indicate more exposed surface of nanosheets than cubic PB. The adsorption capacity of Cs^+^ was about 200 mg/g.

PBA have been used to regenerate nuclear-waste-contaminated soil. Cesium can interact with swelling 2:1 type clay. The oxygen atoms in the inner layer of 2:1 type clay usually binds with potassium. Cesium can replace potassium and interact with clay minerals, resulting in almost irreversible contamination of the soil [55]. First, is necessary to release Cs^+^ from the clay, and then to adsorb the released Cs^+^. Qian et al. have used ionized chitosan that, similar to other cationic polyelectrolytes, increases the interlayer spacing of the clay, allowing desorption of Cs^+^. Then, they utilized PBA functionalized magnetic microgels to adsorb Cs^+^ from the solution after the treatment [56]. The microgels could be regenerated using NaOH (0.1 M) as desorb agent and recycled magnetically while keeping the adsorption capacity constant (149.70 mg/g) after multiple times of use. 

**Table 1:** Comparison among the most relevant adsorption properties of hybrid structures of magnetic Prussian blue nanoparticles.

Adsorbent	Synthesis	Adsorption Capacity/mg g^−1^	Removal Efficiency/%	Equilibrium Time	Kinetic Model	Ref.
Nanoclusters	Single-precursor	45.87	>99.7	6 h	Langmuir	[49]
Nanoparticles	Single precursor	96.00		24 h	Langmuir	[45]
Nanocomposites with graphene oxide	Anchoring the magnetic PB onto the graphene surface	55.56	>90.0	12 h	Langmuir	[46]
Nanoparticles with PDDA as interlayer	Single precursor	16.20	91.0	1 h	Freundlich	[43]
	Co-precipitation		94.0	3 h		[48]
	Co-precipitation		84.7–86.7			[44]
Nanocomposites	Co-precipitation	280.82		24 h	Temkin	[47]
Nanoparticles with PEG	Hydrothermal	274.70	64.8	1 h		[50]
Microparticles	Hydrothermal	16.30	97.0	10 min	Freundlich	[51]
Microgels	Ligand substitution reaction	149.70	83.7	24 h	Langmuir	[56]

The reported studies about the adsorption of Cs by PB or PBA coincide in that this adsorption is a highly selective process that can be described by a pseudo-second-order kinetic model; this implies that the Cs^+^ was chemisorbed and the adsorption rate of the PB composite depended on the active sites rather than the concentration of Cs^+^ in the solution [39]. The equilibrium adsorption isotherm process can be explained in most of the cases by the Langmuir adsorption isotherm model [57]. However, a few studies pointed out that either the Freundlich adsorption isotherm model [58] or the Temkin model [59] agreed with the obtained isotherm [47,60]. Having in mind that the adsorption process is depending on many factors (pH, adsorbent dose or equilibrium time, among others), such discrepancies do not appear as strange. 

The major weak point of PB is its instability in alkaline solutions, which causes hazardous cyanide contamination [5,61]. The maximal concentration of total cyanide in drinking water is 0.07 mg/L according the guidelines established by WHO. To convert PB stable in alkaline solutions, Manabe et al. have added CuSO_4_ to PB so that divalent copper ions act as a shielding element at a high concentration of hydroxide ions [62].

**Figure 3:**
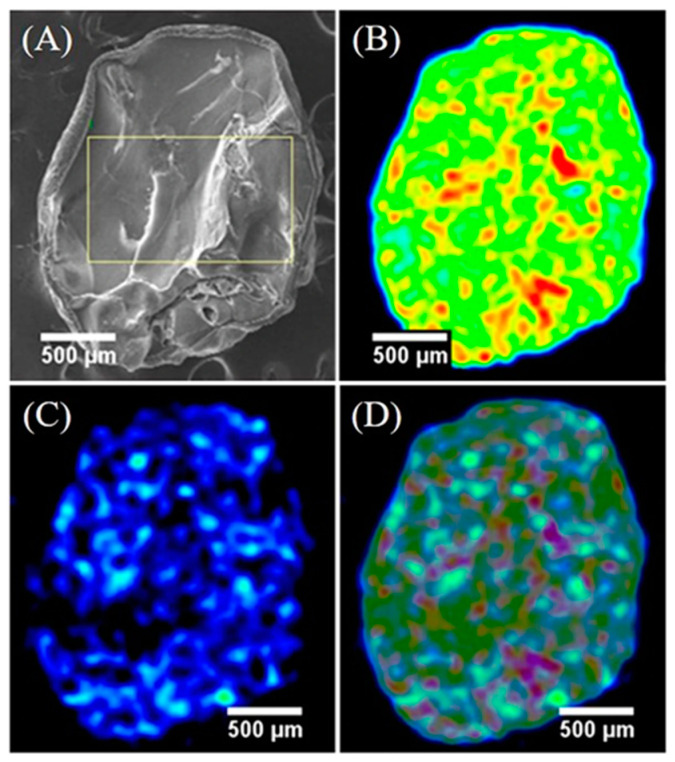
Cesium distribution on PB/cellulose aerogel after adsorption. (**A**) SEM image of the cryo-fractured PB composite. (**B**) EDS mapping of iron. (**C**) EDS mapping of cesium. (**D**) EDS mapping of iron–cesium overlay. Reproduced with permission from [39].

Lee et al. observed that the Cs^+^ were completely diffused onto the PB composite (aerogel formed by cellulose) [39]. This is a prerequisite for a good absorbent material. Figure 3 shows the elemental mapping of the composite after the adsorption of 0.1 ppm Cs^+^ in distilled water for 8 h.

Concerning the PBA, Wang et al. reported on electroactive nanospheres composed of magnetite nanosphere and electroactive cupric hexacyanoferrate coating [60]. The nanospheres could be regenerated by simply switching the potential of the magnetic electrode in a novel electromagnetic coupling regeneration system. The nanosystem showed relatively fast kinetics (the equilibrium was reached in approximately 60 min) and high adsorption capacities toward Cs^+^ (66–105 mg/g). This capacity is nearly unchanged after 20 adsorption–regeneration cycles and a high regeneration efficiency greater than 97% was maintained in each cycle.

### 4.2. Adsorption of Thallium

Thallium (Tl) is a rare metal with high toxicity. Whereas the elemental form of thallium has essentially no toxicity, its univalent (thallous, Tl^+1^) and trivalent (thallic, Tl^3+^) salts are highly toxic. The human body does not discriminate the uptake of Tl^+1^ over K^+^ as the ionic radius of Tl^+^ is similar to that of K^+^, which has fatal consequences because Tl disrupts the proper functioning of the K^+^ involved in many biochemical reactions [63]. In this way, Tl could cause acute and chronic poisoning that would imply degenerative changes in the heart, liver, and kidney. The poisoning with Tl could be caused by the explosion of a “dirty bomb” prepared with radioactive isotopes including cesium and thallium. The presence of nonradioactive Tl in the environment is due to the emission of the metal by coal combustion, cement plants, and nonferrous metals metallurgy. The predominant contamination source of Tl is wastewater from the mining, beneficiation, and smelting of Tl-containing sulfide ores. 

A great number of methods (precipitation, flotation, electrochemical deposition, and solvent extraction) have been developed to remove Tl from industrial wastewater [64]. However, adsorption exhibits great potential due to the advantages of high purification efficiency, low energy consumption, and environmental friendliness [65]. In this way, adsorptive technologies based on the use of PB and PBA have been used. The medication Radiogardasse, prescribed for the treatment of known or suspected internal contamination with radioactive Cs^+^, is also indicated for the treatment of the poisoning by radioactive or nonradioactive Tl. 

Yang et al. determined the relationship between physicochemical properties of insoluble PB and its binding capacity [66]. Meanwhile, by using solutions with different pH, they evaluated the effect of pH and storage conditions on the binding to PB. The results indicated that the hydration state of PB influenced the Tl uptake. The PB with 17 mol of water had a binding rate constant of 0.52, which was reduced to 0.32 when PB was dehydrated to 2.5 mol of water. Later [67], the same group determined that insoluble and soluble PBA, with similar quality attributes, had nearly identical binding capacities. Sangvanich et al. evaluated the removal of Tl^+1^ by PB and Cu(II)ferrocyanide, a PBA, immobilized on mesoporous silica in varied aqueous systems [68]. Compared to PB, the PBA showed higher capacity of adsorbing Tl (28.3 mg/g for PBA and 5.82 mg/g for PB). According to the results, the distribution coefficient of Tl on both sorbents increased with the increase of the pH of the solution. Therefore, the cation ion exchange might be the major adsorption mechanism. This mechanism was also postulated by Vincent el al. [69]: Tl was exchanged with hydrogen ions in the crystal lattice of PB, or with alkali metal impurities bound to PB during the synthesis process. 

### 4.3. Adsorption of Cations

In addition to Cs^+^ or Tl^+^, PB also adsorbs other monovalent and divalent cations. Such cations can compete in a lesser extent with Cs^+^ or Tl^+^ by the exchange with hydrogen ions present into the crystal lattice of PB. The distribution coefficient of Cs on magnetic PB was decreased by the presence of other cations, where K^+^ has the most detrimental effect. The similitude between the Stokes radii of both cations (Cs^+^, 0.119 nm and K^+^, 0.125 nm) or even the hydration radii (Cs^+^, 0.325 nm and K^+^, 0.330 nm) could explain such competition. At this point, it would be interesting to think of using PB as sorbent for K^+^ in patients with hyperkalemia. The competition produced by Na^+^ is much smaller. Vafakhah et al. developed an efficient hybrid capacitive deionization system for removal of NaCl from brackish water, in which PB embedded in a highly conductive reduced graphene oxide aerogel was used as a binder-free intercalation anode to remove Na^+^ ions. The combination of redox-active PB and the three-dimensional porous graphene network yielded a high salt removal capacity of 130 mg/g at the current density of 100 mA/g [70]. 

Although the size of divalent cations is quite different from that of Cs, in absence of Tl^+^ or Cs^+^, PB can adsorb divalent cations, such as Cu^2+^, Co^2+^, Ni^2+^, and Pb^2+^ [71]. By using magnetic PB, Uogintė et al. showed the high capacity of sorption: copper, 138 mg/g, cobalt, 111 mg/g, nickel, 155 mg/g, and Pb, 778 mg/g. It is important to remark the capacity of adsorption of PB by Cu^2+^. This ion forms a high-affinity complex with the β-amyloid peptide, whose accumulation is observed in Alzheimer’s disease [72]. Furthermore, it could exist a relationship between the presence of copper ion and the formation of plaques of β-amyloid. 

### 4.4. Adsorption of Gases 

PB (and especially PBA) offer great potential for gas storage. Long and coworkers explored by first time the gas adsorption properties of PB. They used this material for hydrogen storage at 77 K [73]. Another gas that can be adsorbed into PB is CO_2_. In this regard, CO_2_ capture and removal from a flue gas stream is a technical challenge because of the low concentration of CO_2_ (<15%) present in the gas stream. Thallapally et al. used several PBA to capture CO_2_ and other gases (SO_2_, N_2_, NO, H_2_S). The PBA used adsorbed 8–10% of CO_2_ at room temperature and 1 bar of pressure [74]. The adsorbed amount increased at higher pressures. Moreover, they demonstrated that the materials did not decompose after exposure to the gases. Karadas et al. extended the studies of the CO_2_ adsorption into PBA up to 50 bar. The water molecules present in vacancies of the crystal structure of PB can be eliminated through heating without disrupting the crystal structure, and thus leaving holes available for CO_2_ molecules. However, the CO_2_ adsorption isotherms showed sudden changes in the 35–40 bar range [75].

Ammonia gas released from agriculture and livestock farms is one of the main precursors of fine particulate matter that represents a source of illness affecting the lung and the heart. For this reason, as ammonia is water-soluble, its presence in water is important to the health. As the concentration of Cs and Tl in environmental water is usually negligible, PB and PBA can be used for recovering dissolved ammonia [76]. However, ammonia removal is usually faced with selectivity concerns because the application area often contains chemical fertilizers or biomass matrices, making selectivity an important factor when choosing the adsorbent. Parajuli et al. synthesized a PBA, copper hexacyanoferrate, capable of capturing dissolved NH_4_^+^ and NH_3_ simultaneously in the presence of high concentrations of potassium [77]. Zhang et al. have prepared a PBA, a sodium salt of cobalt hexacyanoferrate, with an adsorption capacity for NH_4_^+^ higher than that of K^+^ and Cs^+^ [78]. These important differences between PB and the indicated PBA have their origin in the switch of the metal in the ligand, the coordination metal, and the monovalent ion, that modify the crystal parameters. More recently, Takahashi et al. have showed that the cobalt hexacyanoferrate can be recycled through water flushing [79]. 

### 4.5. Adsorption of Molecules 

PBA have showed to be able of adsorbing substances different than cations (and ammonia), i.e., capturing gaseous molecules, such as H_2_ or CO_2_ [75,80], or for propane/propylene separation [81]. Boudjema et al. have reported the preparation of several PBA; one of them, with cobalt as transition-metal ion, was able to separate a hydrocarbon mixture (n-pentane, n-hexane, cyclohexane, and cyclohexene) in a humid atmosphere [82]. These results emphasize the remarkable potential of PBA for a gas separation.

**Figure 4:**
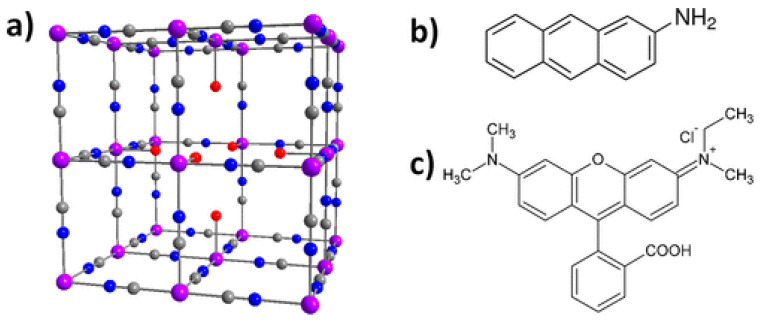
*(***a**) PB showing a single hexacyanoferrate vacancy in the center; (**b**) chemical structure of AA; (**c**) chemical structure of RhB. Color code: purple, Fe (II) and Fe (III); blue, N; gray, C; red, O. Reproduced with permission from [75].

The adsorption by PB/PBA has other applications than those intended to remove molecules or cations from an environment. Larionova’s group rendered magnetic and nonmagnetic PBA nanoparticles luminescent by a synthetic functionalization within the internal porosity of the cyano-bridged framework using 2-aminoanthracene (AA) or rhodamine B (RhB) fluorophore to yield a luminescent system (bifunctional when the nanoparticles were magnetic) [3,83]. Figure 4 shows the two luminophores, which differ in their size, although both are planar. The existence of a single vacancy in the center of PB allows AA to be accommodate inside. RhB is too large to enter within the porosity and it is bounded to the nanoparticle surface. In any case, these nanoprobes could be efficiently used to monitor the cell internalization of the PB nanoparticles by fluorescence imaging.

## 5. Conclusions and Outlook

This review provides a report of the zeolitic properties of PB and PBA, namely its capacity of capturing ions and molecules. As PB is biodegradable and nontoxic, it has been successfully used in medicine or in protection of environment as antidote. The main adsorptive application of PB is to remove cesium and thallium ions. However, in absence of these ions, which do not abound under normal conditions, PB can trap potassium, and to a lesser extent, sodium ions. The substitution of iron cations by other cations in PBA results in a higher versatility in adsorption properties, and these compounds can adsorb gases, ammonia, water, and hydrocarbons. Interestingly, new strategies have focused on the reuse of PB/PBA, affording an environmentally friendly system. 

To date, several research studies have been conducted concerning the adsorptive properties of new PBA, but these properties have not been systematically studied. Thus, more careful studies are required to explore the possibility of a common pattern in the adsorption process and to improve the distribution coefficient. The research conducted with PB is wider, but it presents in general an evident limitation if the application in live beings is considered: much of the research to date has been conducted in laboratory settings, and the interfering effect of food or other biological components on the adsorption has not been still determined. Concerning the adsorption of divalent cations and the chelating effect of suspiciously toxic ions, a systematic research in the presence or absence of monovalent cations is necessary to determine the way of capturing the divalent ions.
